# Ubiquitin family as novel protein partners for molecular glues

**DOI:** 10.1038/s44386-026-00059-0

**Published:** 2026-08-04

**Authors:** Laura Silvian

**Affiliations:** Department of Synthetic Molecular Design, Biogen Inc., Cambridge, MA USA

**Keywords:** Biochemistry, Biophysics, Chemical biology, Computational biology and bioinformatics, Drug discovery, Structural biology

## Abstract

Molecular glues are small molecules that induce ternary complexes between a protein of interest and a partner protein, creating therapeutic mechanisms distinct from traditional inhibitors. This minireview examines binding-site architectures of clinically tested molecular glues and extends these insights to ubiquitin-family partner proteins. Structural analysis suggests recurring recognition features, while emerging computational methods may accelerate discovery and optimization of ubiquitin-directed glues for therapeutic applications.

## Introduction

Molecular glues (MGs) are small molecules that bring a protein of interest (POI) into proximity with a partner protein, resulting in either gain or loss of biological function depending on context. This modality is gaining traction in drug discovery because its downstream cellular effects can be more durable than those of conventional small-molecule inhibitors and because it can expand the range of targetable proteins. The term “molecular glue” was coined by Stuart Schreiber in 1992 to describe an immunophilin–ligand complex that targets calcineurin^1^. The historic discovery of molecular glues and other chemical inducers of proximity, such as PROTACs, has been reviewed recently^2^. Neo surfaces created by molecular glues may enable targeting of proteins previously considered “undruggable” by creating new crevices on the target surface interface once the partner is engaged^3^. The recruited partner protein can have enzymatic function, for example, an E3 ligase that carries out ubiquitination leading to protein degradation. Alternatively, a glue can recruit a non-enzymatic partner and act as a shield to block functional protein–protein interactions or redirect the POI to a different cellular compartment^3^. Both types of molecular glues have entered the clinic^4,5^ (Table 1), motivating efforts to identify glue-binding sites more quickly and systematically.

**Table 1:** Molecular glues in the clinic for different indications

Molecular glue	Company	Current Phase	Indication(s)	Timeframe	Mechanism of Action (MoA)
**Degradative**					
Mezigdomide (BMS-986348/CC-92480)	Bristol Myers Squibb	Phase III	Multiple myeloma	Phase I started 2023	Apoptosis stimulant; CRBN modulator; IKZF1/3 degradation
Golcadomide (BMS-986369/CC-99282)	Bristol Myers Squibb	Phase III/I	non-Hodgkin lymphoma, diffuse large B-cell lymphoma, and follicular lymphoma.	Phase I started 2023	CRBN modulator; IKZF1/3 degradation
Iberdomide (CC-220)	Bristol Myers Squibb	Phase III/I	Relapsed or refractory Multiple myeloma (RRMM), Kidney impairment	Phase I started 2021 Feb 2026 FDA accepted NDA for iberdomide combined with standard treatment (daratumumab + dexamethasone - IberDd) in patients with RRMM.	CRBN modulator; IKZF1/3 degradation
SP-3164	Salarius Pharmaceuticals	Phase I	Non-Hodgkin’s lymphoma and DLBCL	Phase I started in 2024	CRBN modulator: IKZF1 degraders
MRT-2359	Monte Rosa Therapeutics	Phase II/I	Solid tumors, AR mutations	Phase I started 2022	CRBN modulator: GSPT1 degradation for Myc-driven tumors
MRT-6160	Monte Rosa Therapeutics Inc./Novartis	Phase I	Immune-mediated diseases	Phase I started in 2025	CRBN modulator: VAV1 degradation
MRT-8102	Monte Rosa Therapeutics Inc.	Phase I	Inflammatory diseases related to NLRP3, IL1B and IL-6 dysregulation, cardiovascular cardiometabolic	Phase I started in 2025	CRBN modulator: NEK7 degradation
**Non-degradative**					
Cyclosporine (Sandimmune)	Sandoz	approved	Prevent organ transplant rejection	1983 approval	Binds to cyclophilin A and inhibits calcineurin in lymphocytes
Rapamycin (Sirolimus, Rapamune)	Pfizer	approved	Renal transplantation, Lymphangioleiomyomatosis (a rare lung disease)	1999 approval 2015 approval	Binds FKBP to mTORC1 and prevents substrate binding
Daroxonrasib (RMC-6236)	Revolution Medicines	Phase III completed 2026	4 Phase III: three in Previously Treated Metastatic Pancreatic Ductal Adenocarcinoma (PDAC), and one in non-small cell lung cancer (NSCLC)	Phase III started 2024 April 2026: daraxonrasib demonstrated a median OS of 13.2 months versus 6.7 months for chemotherapy	CyclophilinA MG: GTP-bound KRAS (ON)
Elironrasib (RMC-6291)	Revolution Medicines	Phase II/I	Solid tumors	Phase 1 started 2024; 2025 received breakthrough therapy designation for non–small cell lung cancer (NSCLC) harboring *KRAS* G12C mutations who have been previously treated with chemotherapy and immunotherapy and are naive to a KRAS G12C inhibitor.	Covalent cyclophilin A MG: GTP-bound KRAS G12C (OFF)
Zoldonrasib (RMC-9805)	Revolution Medicines	Phase I	Solid tumors, NSCLC	Phase I started 2024; 2026 FDA breakthrough therapy designation for zoldonrasib for non-small cell lung cancer (NSCLC) who have been previously treated with anti-PD-1/PD-L1 therapy and platinum-based chemotherapy	Cyclophilin A MG: GTP-bound KRAS G12D (ON);
ST-01156	SEED Therapeutics	Phase I	Solid malignancies: Ewing sarcoma	Phase I started in 2025; Orphan Drug and Rare Pediatric disease designation	DCAF15 modulator:RBM39 degrader Brain penetrant
NST-628	Nested Therapeutics	Phase I	Solid tumors	Phase I started in 2024	MEK/RAF MG; prevents formation of BRAF-CRAF heterodimers; brain penetrant
IK-595	Ikena Oncology	Phase I	RAS- or RAF-altered advanced solid tumors	Phase I started in 2023, completed in 2025	MEK/RAF MG, inhibits MEK recycling

### Molecular glues vs. bifunctional small molecules

Molecular glues (MGs) and bifunctional molecules both bring two proteins together, but they differ in ease of design and in concentration–response behavior. Bifunctional compounds contain two distinct binding moieties that independently engage each protein; as a result, they are often easier to design by linking two known binders. These molecules can be relatively large (often 800–1000 Da) and can show a “hook effect” or a U-shape in concentration-response curves: at higher MG concentrations, the POI and partner protein become saturated, reducing ternary-complex formation.

MGs are smaller molecules (MW < 500 Da) and often have more favorable absorption, distribution, and metabolic properties with less formulation challenges. MGs may bind weakly to each protein individually but promote cooperative binding that stabilizes the ternary complex. This modality is harder to discover rationally because potency depends on conformational adaptation between protein surfaces. Glues can stabilize otherwise transient protein–protein interactions or create new interactions that are not experimentally observed in the absence of compound. Pharmacologically, molecular glues often exhibit a monotonic concentration–response without a hook effect, reflecting sustained ternary-complex formation at higher compound concentrations^6^.

### Cooperativity of protein–protein interactions and definition of “presenter protein”

Molecular glue hits can be optimized to improve ternary-complex stability as measured by the quantitative cooperativity term (alpha). This is defined as the ratio of the dissociation rate of the binary complex divided by the dissociation rate of the ternary complex. If alpha is high, peak activity tends to span a wider concentration range and the “hook” is wider; if alpha is low, peak activity tends to span a narrower range and the “hook” is narrower.

A “presenter protein” is a partner protein that can bind the molecular glue first, creating a remodeled surface that enhances affinity for the protein of interest^2^. This term is borrowed from immunology by analogy to the major histocompatibility complex (MHC), which “presents” peptide antigens to engage the T-cell receptor. The molecular glue that first engages the presenter protein can exhibit high cooperativity toward the POI.

Non-degradative partner proteins should be versatile binders and highly abundant in relevant tissues to enable occupancy-driven pharmacology. For example, FKBP12 employs a surface loop for recognition and is remarkably versatile, enabling the formation of high specificity, highly cooperative complexes with many distinct targets using different repertoires of amino acid residues^7^. FKBP12 is also highly abundant and widely distributed in different tissues^8^. The concept of using a “super” presenter protein to block a receptor or sequester it is being pursued by Magnet Biomedicine, which claim to have developed a strategic and systematic way of selecting intracellular and extracellular partner proteins in a tissue-specific manner for a target^9^.

Molecular glues that bind cooperatively can be identified through a selection binding experiment involving DNA-encoded libraries (DELs). In a DEL selection experiment, small molecules are tagged with a DNA barcode for ease of deconvolution, and the tagged POI is attached to a bead for pull-down. To identify an MG, the partner protein is also added to enable ternary-complex formation. In an innovative study, the authors used a method of changing the presenter ratio as a predictive measure of cooperativity when searching for a compound that would bridge the bromodomain BRD9 and the VHL-elongin C -elongin B (VCB) complex^10^. The bead-immobilized BRD9 was added to either the DEL library alone (binary complex) or to a DEL library that had been preincubated with tag-less VCB at different presenter:POI ratios. Compounds that were identified under high presenter:POI ratios showed no hook effect, suggesting they were more cooperative.

### Partner protein classes

Partner proteins for molecular glues span a variety of protein classes and can drive different cellular outcomes, including targeted protein degradation, targeted post-translational modification, targeted protein stabilization, and targeted protein inhibition^11^. For simplicity, we highlight partner-protein classes and mechanisms of recognition that are currently being explored in clinical trials, with the expectation that additional partner classes will be investigated in the future. In particular, structural features used for recognition by different partner-protein classes are described to illustrate the range of interactions that molecular glues can mediate.

Cullin E3 ligases are widely used degradative partner proteins that ubiquitinate the protein of interest and direct it for proteasomal degradation. E3 ligases used for molecular glues include Cereblon (CRBN), Von Hippel-Lindau tumor suppressor modulator (VHL), Ring finger protein 126 (RNF126), DNA damage-binding protein 1 (DDB1), DDB1 and CUL4 associated factor 16 (DCAF16) and others. One of the most adaptive E3 ligases is CRBN, and the mechanisms by which it recognizes neo substrates are diverse. Glued substrates have included those containing canonical β-hairpin glycine-rich loops (CK1a, WEE1, CK1d, NEK7, etc.), helical glycine-containing loops (Vav1, mTOR), and substrates lacking these features (TBK1, G3BP2), as reviewed recently^12,13^. Von Hippel-Lindau (VHL) is another E3 ligase that is widely used in PROTACs and has recently been exploited for molecular glues. An example of a VHL molecular glue is a degrader for cysteine dioxygenase (CDO1) identified by Novartis^14^.

A rational design strategy is to affix a specific chemical handle on either the POI or partner protein and diversify its solvent-facing region to enable the ternary complex formation. For example, diverse moieties were appended to the exit vector of handles to the RING E3 ligase RNF126. Depending on the chemical affixed, these molecules were able to hook RNF126 to multiple POIs including LRRK2, BRD4, BTK, and others^15^. Similarly, a chemical handle binding to the active site of CDK12 kinase was used to recruit the DNA damage binding protein 1 (DDB1) E3 ligase adaptor and indirectly degrade the cyclinK that was complexed with CDK12. In these structures the compound is positioned at the DDB1-CDK12 interface and enables the E3 ligase RBX1 to ubiquitinate it^16^.Aryl sulfonamide handles for the RING E3 ligase DCAF15 has been exploited by E7820, indisulam and tasisulam to degrade the splicing factor RBM39 for MYC-driven cancers with aberrant alternative pre-mRNA splicing^17^.

The handle can also be a reversible-covalent warhead, which has been described for DCAF11 and DCAF16. Using a ‘template-assisted covalent modification mechanism’ molecular glues that covalent link to a DCAF16 Cys58 were shown to degrade BRD4^18^. The cryo-EM structure of the DCAF16, BRD4 and an intramolecular bivalent glue shows that the molecular glue covalently binds DCAF16 and also engages two adjacent bromodomains of the BRD4 target protein in cis, enabling a rationally designed, specific interaction with this POI at the interface of the two BRD4 bromodomains which rearrange to form the ternary complex^19^. DCAF16 has been used as the partner protein for a reversible covalent glue targeting BRD4 or BRD9 by Amphista, in which the molecular glue engages surface Cys58 of DCAF16^20,21^.

Some glues work by preventing an enzyme’s catalytic turnover or by redirecting the POI to bind to a bystander protein. For example, an MG was created between RAF and MEK kinases that disrupts a dysregulated Ras/Raf/MEK/ERK signaling pathway in cancer. IK-595 traps MEK in an inactive complex with any RAF isoform and enables prolonged pathway inhibition^22,23^. Other non-degradative molecular-glue partners include 14-3-3 proteins, which enhance binding to short phospho-Ser/Thr motifs in intrinsically disordered regions of client proteins such as Erα, PIN1, CRAF, and ChREBP, thereby redirecting cellular localization. This is a platform adapted by Ambagon Therapeutics^24^.

One interesting partner class is the immunophilin family, which is targeted by natural-product MGs such as rapamycin and cyclosporin and forms ternary complexes through a multistep process. The molecular glue cyclosporin binds the immunophilin cyclophilin, and this composite surface then binds calcineurin to block its T-cell receptor cytoplasmic mediator function. Neither cyclosporin nor cyclophilin can bind calcineurin individually, making cyclophilin a ‘presenter’ protein for a molecular glue [2].

FKBP12 is a key presenter protein that has been adapted to target many proteins of interest [7]. Rapamycin acts by gluing FKBP12 to the FRB domain of mTORC1 (within the mTOR–Raptor complex). The resulting FKBP12–rapamycin complex sterically blocks access of kinase substrates to the mTORC1 active site. Rapamycin is selective for mTORC1 over mTORC2 because the FRB domain is accessible in mTORC1, whereas in mTORC2 it is occluded by Rictor (rather than Raptor)^25^. As an extension of the serendipitous discovery of rapamycin, a purposeful screen at high concentrations was conducted to identify alternative glues. The authors identified a bicyclic sulfonamide compound (cpd7) that binds in the same hotspot as rapamycin and enhances FKBP12 binding^26^.

Another recent example of repurposing FKBP12 was a study that identified autophagy inducers using a DNA-encoded library screen^27^. ATG16L1 is indispensable for autophagy, but a loss-of-function mutation (T300A) makes it more susceptible to cleavage by caspase 3. The goal of this screen was to use FKBP12 as a shield to bind ATG16L1 and prevent caspase3-mediated cleavage. Compounds that bind the ternary complex of ATG16L1 and FKBP12, but not ATG16L1 alone, were identified.

FKBP12 has been adapted as a blocking presenter protein by the company Rapafusin who have used it to block multiple therapeutic targets including a nucleoside transporter ENT1, whose inhibition by FKBP12 prevents adenosine uptake in cells-- the compound is active in a kidney ischemia reperfusion injury^28^.

In a similar manner, cyclophilin A is a good presenter protein. Molecular glues have been designed to block KRAS from binding to RAF using cyclophilin A (CypA) as a blocking presenter protein. Revolution Medicine originally used CypA to target KRAS G12C by creating macrocyclic peptide molecules that recognize CypA on one side and, on the solvent-exposed side, engage KRAS G12C covalently^29,30^. Later, the cysteine-reactive warhead was replaced by a cyclized tricomplex scaffold that glues wild-type and other variants of KRAS to CypA^31^.

### Molecular glues as approved therapies and ones that have entered the Clinic

#### Degrading molecular glues

While more than 25 bifunctional PROTACs have entered the clinic for prostate, breast, and hematologic cancers as reviewed recently^32^ fewer molecular glues have entered the clinic, but they are noteworthy (Table 1). CRBN modulators Mezigdomide, Golcadomide, and Iberdomide are in Phase III and target Aiolos/Ikaros and ZFP91 for multiple myeloma and hepatic impairment. DCAF15-targeting molecular glues include SEED Therapeutics ST-01156, which targets the splicing factor RBM39 for patients with solid tumors and is in Phase I. Salarius has advanced a glue degrader SP-3164 against IKZF1/3 into Phase I clinical trials^33^. Monte Rosa has advanced a degrader of translation termination factor GSPT1 into Phase I trials (MRT-2359). Beyond oncology applications, Monte Rosa Therapeutics and Novartis have advanced degradative molecular glues into clinical trials targeting inflammatory diseases. Monte Rosa has initiated a Phase I trial of a CRBN-based NEK7 molecular-glue degrader (MRT-8102) and a VAV1 glue degrader (MRT-6160)^34^.

#### Blocking molecular glues

Cyclosporin is an approved molecular glue (since 1983) and is marketed by Sandoz for organ transplant rejection^35^. For cancer, immune, cardiovascular, and integumentary indications, rapamycin-derived compounds (rapalogues) such as Everolimus, Temsirolimus, and RTB101 are approved MG therapies^36^.

Revolution Medicines has recently reported the outcome of a successful Phase III study with a molecular glue Daraxonrasib that targets the pan-RAS (ON) state and significantly extends survival of patients with metastatic pancreatic cancer^37^. They are also following up in Phase I/II with molecular glues Elironrasib (RMC-6291, targeting KRAS G12C)^30^ and Zoldonrasib (targeting the KRAS G12D (ON) state)^31^ for solid tumors (Table 1).

Nested Therapeutics has discovered and developed a molecular glue (NST-628) that binds both MEK and RAF, stabilizing their interaction. By doing so, it inhibits catalytic turnover and also enables MEK to act as a shield that blocks a surface required for BRAF–CRAF heterodimer formation^23^. NST-628 is currently in Phase I for solid tumors. A competitor to Nested is Ikena, who also has started phase I clinical trials with molecular glues for RAF (IK-595)^22^ (Table 1).

#### Ubiquitin family

Given the clinical success of molecular glues, there is renewed interest in identifying intracellular proteins that are sufficiently abundant to serve as partner proteins for redirected biology. Ubiquitin and ubiquitin-like (UBL) family proteins are abundant across human tissues and form transient PPIs, which could signal their potential use as MG partner proteins. In fact, they have served as partner proteins in the serendipitous discovery of molecular-glue tool compounds for Parkin E3 ligase^38^, CDC34A^39,40^, and the COP9 signalosome^41,42^ (Table 2). Because this family engages with many proteins, it is important to ask whether molecular glues bind it through a shared mechanism. Below, we describe conserved surfaces on these small proteins that mediate protein–protein interactions and how these surfaces are augmented by molecular glues.

**Table 2:** Molecular glue compounds that bind to ubiquitin/NEDD8 as a partner protein

Molecular glue	Partner protein	Protein of Interest	Reference
BIO-2007817, BIO-1975900	Phospho-ubiquitin (pUb)	Parkin E3 ligase	^38^
BDC2245574, CC-0651, 2ab	Ubiquitin	CDC34A (E2 enzyme)	^39,40^
CSN5i-3	NEDD8	COP9 signalosome (CSN)	^41,42,56^

Ubiquitin^43^, NEDD8 (Rub1)^44^, and SUMO1^45,46^ are homologous proteins with the β-grasp fold and can modify proteins as monomers or polymerized units. Ubiquitin plays a crucial role in a wide range of biological process and dysregulation of ubiquitination can lead to abormal pathway activation in diseases such as cancer, neurodegenerative diseases and metabolic disorders^47^. NEDD8 is closely related to ubiquitin and enables cullin E3 ligases to assemble properly for ubiquitin transfer^44^. SUMO1 has a similar fold to ubiquitin and regulates a wide variety of cellular processes, including transcription, DNA repair, ubiquitin E3 ligation, and cell-signaling pathways. SUMO1 additionally contains a hydrophobic groove that binds short linear motifs known as SUMO-interacting motifs (SIMs), which are found across a broad spectrum of partners^46^.

The post-translational modification pattern of ubiquitin acts as a signal that can tag proteins for degradation by the proteasome, alter subcellular localization, or modulate interactions with protein or DNA partners. In mono-ubiquitination, a ubiquitin monomer is covalently attached to lysine residues on target proteins through an isopeptide bond formed with ubiquitin’s C-terminal Gly-Gly motif. In poly-ubiquitination, different linkage topologies are formed by coupling each new ubiquitin to different lysines on the mono-ubiquitin tag. These linkages convey different cellular signals—for example, K63-linked ubiquitin chains are not typically associated with protein degradation but instead activate proteins involved in the DNA damage response. Linear ubiquitin chains play a role in regulating inflammatory and immune responses^43,47^.

The 76 amino acids that make up the β-grasp fold are arranged as four β-strands interspersed by one α-helix and two 3–10 helices. The surface of ubiquitin displays hydrophobic patches that mediate interactions with other proteins. One hydrophobic patch is centered around Ile44, Val70, and Leu8, with surrounding electrostatic interactions involving Lys48, Arg42, Arg72, Lys6, and His68 (often termed the Ile44 patch). A second hydrophobic patch is centered around Ile36 and can be considered together with nearby residues toward the C-terminus (often termed the Ile36 patch). When ubiquitin chains form with different linkages, these hydrophobic patches adopt different relative geometries. For example, the K48 di-ubiquitin linkage positions the hydrophobic patches in a more compact arrangement compared to the K63 di-ubiquitin linkage^43^.

**Fig. 1:**
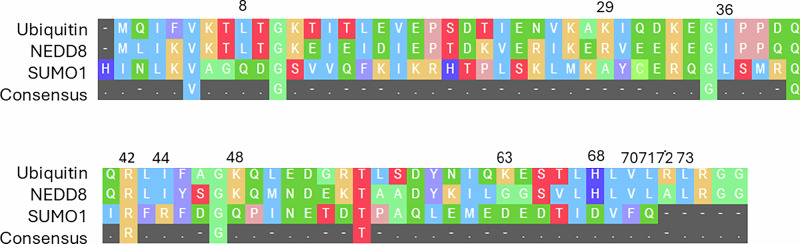
Structure-based sequence alignment of ubiquitin (PDB ID: 8W31), NEDD8 (PDB ID: 9EFV), and SUMO1 (PDB ID: 1WM3), highlighting residues in recognition hotspot positions and their conservation across the Ub-family. These sequences were aligned using the pairwise structure alignment tool hosted on the RCSB website using FATCAT(flexible) as the method (https://rcsb.org/alignment) and FASTA output was entered into Clustal Omega v1.1.0 alignment tool.

NEDD8 is 56% identical to ubiquitin and has the same fold. A structural superposition of ubiquitin (RCSB ID: 1UBQ) and NEDD8 (RCSB ID: 1NDD) using FATCAT2^48^ shows an RMSD of 0.79 Å over 74 residues. All lysines are conserved between ubiquitin and NEDD8 except Lys29 (Asg in NEDD8) and Lys63 (Gly in NEDD8). The Ile44 and Ile36 hydrophobic patches are conserved between ubiquitin and NEDD8 (Fig. 1).

Lack of sequence conservation suggests that SUMO1 will not have similar architecture for interactions with MGs. Using the same alignment method, SUMO1 is 18.4% identical to ubiquitin, has the same fold (RCSB ID: 2N1V), and aligns with ubiquitin (1UBQ) with an RMSD of 1.77 Å over 76 residues. SUMO1 also contains a 20-residue N-terminal extension. The lysine residues that form linkages in ubiquitin are not conserved in SUMO1, nor are the hydrophobic patches. For example, Lys48 is replaced with Gln in SUMO1 and Lys63 is replaced with Asp in SUMO1. Furthermore, hydrophobic patch residues that are conserved between ubiquitin and NEDD8 are not conserved in SUMO1--Ile44 (Arg in SUMO1), Val70 is Gln in SUMO1, Leu8 is Gln, and Ile36 is Leu-- and thus the protein-protein interactions are not transferrable. Thus, while SUMO1 shares the β-grasp fold, it is important to restrict the search for ubiquitin-family molecular glues binding sites to proteins that interact with ubiquitin and NEDD8.

Given the similarity of protein–protein interaction surfaces between ubiquitin and NEDD8, one might expect a shared binding-site theme for molecular glues that mediate interactions between either partner protein and a POI. In the past few years, three cases have been reported in which a molecular glue binds at an interface between ubiquitin (or phospho-ubiquitin) or NEDD8 and a target of interest. Below, we compare these three systems structurally to assess whether a common binding site could be leveraged for rational drug design.

#### Parkin E3 ligase- pUb

A small-molecule activator of the E3 ligase Parkin was identified through a biochemical screen using reconstituted Parkin, E2, E1, and phospho-ubiquitin (pUb) for early-onset Parkinson’s disease. Later, its mechanism of action was determined by X-ray crystal structures, which showed that it is a molecular glue that binds Parkin to phospho-ubiquitin. While several activating compounds were identified, one was improved through structure–activity relationships to achieve sub-micromolar potency in biochemical assays and showed a clear enantiomer preference. While the compound shows enhanced activity in an auto-ubiquitination biochemical assay, binding to full-length Parkin could not be observed. The mechanism of action of BIO-2007817 was determined by measuring binding to a minimal Parkin construct (RORBR domain), in which the N-terminal phospho-ubiquitin-binding region and the C-terminal RING2 domain were removed, in the presence and absence of phospho-ubiquitin. The compound likely could not be observed binding to full-length Parkin because its binding site is occluded by the ACT domain in the fully activated form or by the C-terminal RING2 domain in the autoinhibited form.

**Fig. 2:**
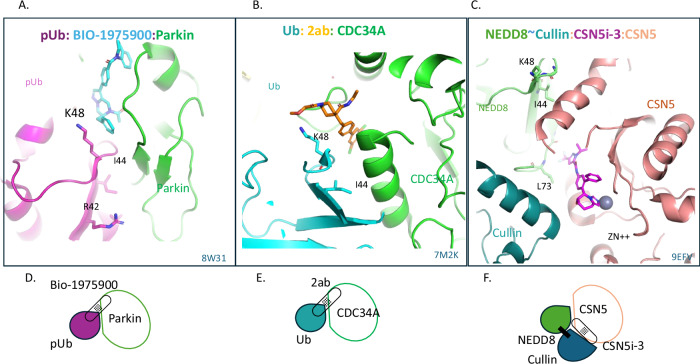
Interface of pUb and Ub with target proteins Parkin or CDC34A and molecular glues. **A** Interaction between Parkin RORB domain(green) and pUb (magenta)with molecular glue BIO-1975900 (blue)(PDB ID: 8W31). **B** Interaction between CDC34A (green) and Ub (cyan) with molecular glue 2ab(orange) (PDB ID: 7M2K). **C** Interaction between CSN5 (pink) of the COP9 signalosome and the NEDD8 (green)~cullin (blue) complex through MG CSN5i-3 (magenta) (PDB ID: 9EFV). Under each structure, a simplified cartoon depicts the glue as a Band-Aid and highlights its location with respect to Ub or NEDD8: **D** Parkin and pUb held by molecular glue BIO-1975900, **E** CDC34A and Ub held by molecular glue 2ab, **F** CSN5 and NEDD8-Cullin held together by molecular glue CSN5i-3. This figure was made using Pymol (https://pymol.org) and the cartoons were made using Powerpoint.

Through mutagenesis, NMR, and X-ray crystallography, the binding site was confirmed to lie mainly in the peptide-binding groove of Parkin, at the interface with phospho-ubiquitin (PDB ID: 8W31). The pUb:RORBR binary construct has an affinity of 1.7 μM, which improves dramatically to 0.7 nM in the presence of the activating molecular glue BIO-2007817. The compound acts as a molecular glue, or “tape,” to knit together the two domains (Fig. 2A). On the phospho-ubiquitin surface, the compound binds mainly to Lys48 on the outer rim of the Ile44 hydrophobic patch, extending the hydrophobic interaction surface.

#### CDC34A-Ub

In a second example using ubiquitin as a partner protein, a recent study further investigated the serendipitous discovery of CC0651, a specific inhibitor of the E2 conjugating enzyme CDC34A. As an E2 enzyme, CDC34A donates activated ubiquitin to the cullin-RING family of E3 ligases. In a high-throughput screen in 2011, CC0651 was discovered as an inhibitor of ubiquitination of p27^KIP1^ by blocking transfer of charged ubiquitin to SCF^SKP2^. The compound potentiated formation of mono-ubiquitinated CDC34A, had a potency of 31 μM, and showed poor cellular efficacy. It was noted that the tool compound binds a surface of CDC34A that interacts with the thioester-linked donor ubiquitin of the E2~Ub complex. Later, in 2014, the authors solved the structure of ubiquitin-linked CDC34A with CC0651 (PDB ID: 4MDK) and demonstrated that the compound binds in an induced composite pocket between ubiquitin and the E2 enzyme, slowing thioester discharge without preventing E3 ligase binding.

In a more deliberate effort, the same authors screened a library of ~10,000 compounds in a proximity assay to measure interaction between CDC34A and ubiquitin. Compound BDC2245574 was identified and shown to be structurally related to CC0651. Using structure-guided exploration of hybrid molecules such as 2ab (PDB ID: 7M2K), the authors improved potency to 2–4 μM in the in vitro proximity assay (Fig. 2B). This compound 2ab showed a similar improvement in the in vitro ubiquitination assay, and the improved potency led to activity in PC3 cells. Like the Parkin activator, this compound binds mainly to the POI (CDC34A) and engages a similar interface on ubiquitin, including the Lys48 side chain. This extends the hydrophobic Ile44 protein–protein interaction hotspot.

#### COP9 signalosome- NEDD8

Lastly, a recent study identified a molecular glue that binds near the cullin domain of an E3 ligase and NEDD8, helping assemble the complex. NEDD8 is covalently bound to the cullin domain, and its removal (de-neddylation) by the COP9 signalosome (CSN) is blocked by the compound CSN5i-3. The COP9 signalosome comprises multiple subunits, including CSN5. A cryo-EM structure of the COP9 signalosome bound to NEDD8-attached cullin and CSN5i-3 shows that this compound acts as a molecular glue at the NEDD8/cullin–CSN5 interface. A strongly cooperative binder, CSN5i-3 binds CSN with only micromolar potency but achieves nM potency in inhibiting de-neddylase activity. The structure shows that the compound not only binds the substrate isopeptide-linkage cavity within the CSN5 domain but also acts as a glue to slow CSN release. It does so by engaging the isopeptide-linked region of NEDD8 (Leu71 and Leu73) (PDB ID: 9E77, 9EFV). In contrast to the Parkin and CDC34A examples, which engage Lys48, this glue engages the C-terminal β-sheet/isopeptide-linked region and similarly extends the hydrophobic interface between NEDD8 and CSN5 (Fig. 2C).

The cellular outcome of each glue is context-dependent and can reflect either gain or loss of function. The enhanced interaction between Parkin and pUb induced by BIO-2007817 activates certain mutant forms of Parkin. The enhanced interaction between CDC34A and ubiquitin induced by BDC2245574 suppresses thioester hydrolysis, leading to inhibition of E3 ligase activity. Lastly, enhanced interaction between CSN5 of the COP9 signalosome and the NEDD8~cullin complex inhibits assembly and disassembly of cullin E3 ligases, dampening the pathway. Thus, molecular glues can either activate or suppress distinct signaling pathways by engaging ubiquitin-family proteins.

#### Rational design applied to Ub-family molecular glues

In the three examples described, the molecular glue does not bind in the center of the ubiquitin–POI interface; instead, it binds at the edge of the hydrophobic interface, increasing the overall buried surface area. For both the Parkin activator and CDC34A, the glue draws most of its binding energy from the POI rather than the partner protein and shows cooperativity of ~1000×. This is partly due to the nature of ubiquitin’s surface, which is relatively flat and hydrophobic, where a lysine can act as a lid over a pocket formed mainly within the POI. As noted above, being able to predict molecular-glue binding that enhances transient protein–protein interactions could enable faster identification of glues for a variety of ubiquitin complexes. After predicting a binding site, improving surface complementarity is the next frontier for optimizing the MG.

Given this shared mechanism, one approach to discover ubiquitin-directed MGs is to scan ubiquitin complexes in the PDB for binding pockets within the POI that are in proximity to Ub Lys48 to allow a compound to extend the hydrophobic interface. Experimentally, ternary-complex screening assays could include the Ub Lys48Ala variant as a negative control for MG binding. If a new ubiquitin-directed MG is identified, which computational approaches could be used to optimize its cooperativity?

### Methods/technologies for predicting molecular glue binding sites

Three methods are described below for predicting how molecular glues might interact within a ternary complex: graph-based approaches, co-folding approaches, and surface-based methods (Table 3). Graph-based approaches model residues as nodes and interactions as edges, enabling docking, interaction-site prediction, and ternary-complex scoring. Co-folding methods use sequence and multiple-sequence-alignment information to predict the 3D structure of two interacting proteins as a co-folding problem; these methods can perform well when large hydrophobic interfaces drive co-folding. Surface-based methods use molecular docking and machine learning to analyze surface shape (geometric and electrostatic features) to identify interaction hotspots and assess interface compatibility.

**Table 3:** Methods/technologies for predicting molecular glue binding sites or improving glue’s potency and cooperativity

Tools for rational design	Program/approach	Strengths	Weaknesses
Lessons from prior examples discovered serendipitously	Use of secondary structure for predicting CRBN substrate binding sites, covalent warheads to DCAF16, common architecture of binding to partner protein	Has the promise to jump start new MG discovery by targeting a particular interaction mode.	Limited to examples that have been discovered, could be improved with proteomics.
Docking software (physics-graph based approach)	FEP+, Glide, etc.	Moderately good method, tried and true method for docking into binary complexes	Assumes no significant backbone conformational changes upon binding of ternary complex.
Co-folding approaches	BOLTZ, alphafold, Chai, Proteinix, RoseTTAFold	Trained on protein-protein interactions within PDB database, which are generally strong interactions, not transient	Currently not sufficient molecular glue neosurfaces in the pdb to train on, hence these approaches will be more successful with more examples of molecular glues in databases.
Surface-based approach to match the interaction surfaces of one protein with the complementary charge and shape of the other protein	MaSIF-NeoSurf	This technique may be useful for flat surfaces and has been successful for some molecular glue degraders, particularly useful for designing mini-proteins that complement epitopes.	Will not be as useful if the surface changes conformation upon binding in ternary complex.

Graph-based approaches use docking software packages such as Glide and free energy perturbation (FEP+) (Schrödinger^49^), or molecular-mechanics methods such as MM-GB/SA^50^, as well as flexible-receptor docking approaches such as ICM-Pro docking (MolSoft)^51^. These methods have been used to rank-order compounds by their predicted ability to promote ternary-complex stability^52^. For Parkin, MM-GB/SA rescoring within the known molecular-glue binding pocket accurately rank-ordered compounds by IC_50_^38^.

At the time of this writing, co-folding approaches for molecular-glue ternary-structure prediction have been unsuccessful. In one trial using AlphaFold 3, Boltz-1, Chai-1, Proteinix, and RoseTTAFold All-Atom, the success rate for identifying protein–protein interactions (~51%) was higher than for identifying native ligand–pocket interactions (<33%)^53^. In a study by Ternary Therapeutics comparing the graph-based approach FEP+ with the co-folding approach Boltz-2 for predicting molecular-glue binding affinity, FEP+ showed better predictivity, with RMSE values of 0.4–1.25 kcal/mol^54^.

Surface-based approaches are one of the fastest-growing areas for identifying binding sites for molecular glues. MaSIF-NeoSurf^55^ uses a geometric deep-learning framework to extract surface-patch descriptors from protein surfaces and match them to complementary protein surfaces in geometry and chemistry. It can generate surface fingerprints trained on protein–protein interactions and apply them to neo surfaces partly composed of small molecules, enabling design of de novo mini-protein binders. The authors report identification of mini-protein binders against BCL2–venetoclax, DB3–progesterone, and PDF1–actinonin neo surfaces. More broadly, generating fingerprints of neo surfaces from existing structures in the PDB and matching them based on electrostatics and shape complementarity may be useful for predicting molecular-glue binding at protein–protein interfaces.

### Conclusions

Molecular glues are increasingly used in drug discovery because they extend cellular responses longer than traditional inhibitors. Several molecular glues are advancing through the clinic for cancer, organ transplant, and other indications. New molecular glue compounds may be more discoverable if we know where to look and how to screen for them. Recent examples of ubiquitin-family proteins serving as partner proteins for molecular glues point to opportunities for future exploration in using ubiquitin to rewire cellular signaling. Ubiquitin-family members have several hallmarks of privileged partner proteins: they are highly abundant in many tissues and binding to ubiquitin alone may be less likely to produce negative effects. Unlike FKBP12, Ubiquitin does not use a loop as a flexible recognition motif that adapts to different surfaces^7^. Instead, this protein binds via common hydrophobic hotspot residues on a beta sheet. It can interact with molecular glues at the edge of this hydrophobic surface- using the Lys48 sidechain or near the iso-peptide linkage. The MG mostly interacts within a pocket of the POI, but the additional interaction with Ubiquitin can extend the half-life of the protein-protein complex. Due to the small number of examples of MG that interacts with the Ub-family we may only be observing the tip of the iceberg of possible MG binding poses.

The same computational tools used to design and optimize traditional small-molecule drugs can also be applied to enhance the potency of molecular glues at protein–protein interfaces. Proteomics, high-resolution structural methods such as cryo-EM to elucidate larger complexes, and improved docking and scoring algorithms all contribute to enabling more rational discovery and optimization of molecular glues. The discovery of molecular glues for therapeutic purposes is likely to grow exponentially in the next few years, and the molecular recognition mechanisms that MGs exploit will become more predictable.

## Data Availability

The structural data used to analyze the binding site of Molecular Glues that utilize Ubiquitin family members as partner proteins were from the RCSB protein databank and have the following references: Parkin/pUb Molecular glue (PDBID 8W31), CD34A/Ub Molecular glues (PDBID 4MDK, 7M2K) and Cullin/CSN5/NEDD8 Molecular glue (PDBID 9E77, 9EFV).
